# CD4+FOXP3+ Regulatory T-Cell Subsets in Human Immunodeficiency Virus Infection

**DOI:** 10.3389/fimmu.2013.00215

**Published:** 2013-07-30

**Authors:** Federico Simonetta, Christine Bourgeois

**Affiliations:** ^1^INSERM, U1012, Le Kremlin-Bicêtre, France; ^2^Université Paris-SUD, UMR-S1012, Le Kremlin-Bicêtre, France; ^3^Division of Immunology and Allergy, Department of Internal Medicine, Geneva University Hospitals, Geneva, Switzerland

**Keywords:** HIV, Treg, FOXP3, CD25

## Abstract

The role of CD4+FOXP3+ regulatory T cells (Treg) in human immunodeficiency virus (HIV) infection has been an area of intensive investigation and remains a matter of ardent debate. Investigation and interpretation suffered from uncertainties concerning Treg quantification. Firstly, Treg quantification and function in HIV infection remain controversial in part because of the lack of reliable and specific markers to identify human Tregs. Secondly, analyzing Treg percentages or absolute numbers led to apparent discrepancies that are now solved: it is now commonly accepted that Treg are targets of HIV infection, but are preferentially preserved compared to conventional CD4 T cells. Moreover, the duality of immune defects associated to HIV infection, i.e., low grade chronic inflammation and defects in HIV-specific responses also casts doubts on the potential impact of Treg on HIV infection. Tregs may be beneficial or/and detrimental to the control of HIV infection by suppressing chronic inflammation or HIV-specific responses respectively. Indeed both effects of Treg suppression have been described in HIV infection. The discovery in recent years of the existence of phenotypically and functionally distinct human CD4+FOXP3+ Treg subsets may provide a unique opportunity to reconcile these contrasting results. It is tempting to speculate that different Treg subsets exert these different suppressive effects. This review summarizes available data concerning Treg fate during HIV infection when considering Treg globally or as subsets. We discuss how the identification of naïve and effector Treg subsets modulates our understanding of Treg biology during HIV infection and the potential impact of HIV infection on mechanisms governing peripheral differentiation of adaptive Tregs.

## Introduction

CD4+FOXP3+ regulatory T cells (Treg) are a critical CD4 T-cell subset involved in the control of immune-tolerance by regulating immune-homeostasis and limiting immune-activation. Defects in Treg cell numbers or function have been related to the development of human autoimmune diseases, while increases in Treg numbers or activity could limit anti-tumoral immune-responses. In contrast to these two scenarios, in which beneficial or detrimental roles of Treg are easily predictable, a much more complex picture emerges for the role of Treg in infectious diseases. Treg mediated inhibition of antimicrobial immune-responses could lead to ineffective clearance of the pathogen contributing to the chronicization of the infection. On the other side, Treg participate to terminate immune-responses thus preventing exacerbated and potentially harmful immune-activation (1, 2). The impact of Treg during human immunodeficiency virus (HIV) infection is even more difficult to integrate due to the dual features of HIV infection. HIV infection is a chronic viral infection inducing drastic CD4 T-cell depletion that is associated with both immune deficiency and dysregulated chronic immune-activation. The control of viral replication is highly dependent on HIV-specific T-cell responses as revealed by the analysis of patients who spontaneously control the virus. Chronic inflammation sustains CD4+ T-cell decay and participates to loss of functional CD8 T-cell activity. Improving HIV-specific responses and limiting chronic immune inflammation are current goals of HIV therapies. Theoretically, Treg may suppress both chronic immune inflammation and HIV-specific responses being thus both beneficial and deleterious in HIV pathogenesis (3, 4). Whereas the dual role of Treg in the pathogenesis of HIV infection is now accepted, debates are still vivid to determine whether residual Treg exert these dual effects simultaneously or sequentially. Interestingly, Treg inhibition does not solely apply to immune functions, but also to the virus: Treg have also been described to directly inhibit HIV infection and replication. Finally additional complexity emerges from the observation that Treg may constitute a potent reservoir, thus leading to consider infected Tregs as an obstacle to efficient control of HIV infection. Determining the net impact of Treg cells on HIV infection remains a matter of ardent debate and face major hurdles: (a) Treg quantification during HIV infection remains controversial in part because of the lack of reliable and specific markers to identify human Treg; (b) expression of Treg quantification using percentages or absolute numbers led to obvious discrepancies that need to be discussed; (c) HIV infection recovers multiple immunological and viral status including patients during primary infection, chronically viremic patients, aviremic antiretroviral therapy (ART) treated patients or spontaneous controllers, which should be considered independently; (d) the accuracy of Treg analysis in the blood versus crucial sites such as lymph-node or gut associated mucosa is also under debate; (e) Treg have been essentially identified so far as a unique population whereas increasing evidence demonstrate high diversity in function and ontogeny. One may also question whether different Treg subsets may differently modulate HIV pathogenesis. Integrating Treg heterogeneity may prove crucial to dissect the impact of Treg mediated suppression during HIV infection. In the present review we will focus on Treg phenotypic and functional heterogeneity in order to elucidate Treg fate during HIV infection and to better delineate the protective or pathogenic roles of Treg cells in HIV infection.

## HIV Infection and Global Tregs

### Identification strategies

**Figure 1:**
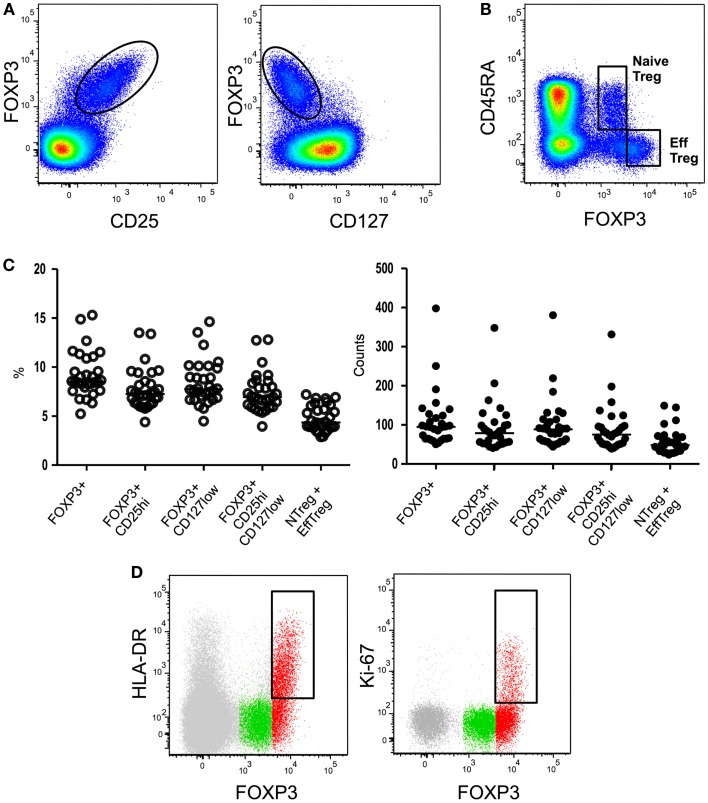
**Flow cytometryidentification strategies of Treg subsets**. **(A)** Global Treg identification based on FOXP3, CD25, and CD127 expression by CD4 T cells. **(B)** Expression of CD45RA and FOXP3 by CD4 T cells allows the identification of CD45RA+ FOXP3low resting or naïve Treg and CD45RA- FOXP3high activated or effector Treg. **(C)** Treg proportions and absolute numbers obtained employing different existing gating strategies. **(D)** Further identification of effector Treg subsets based on HLA-DR or Ki67 expression. HLA-DR or Ki67 expression in global CD4 T cells (gray), CD45RA+ FOXP3low naïve Treg (green), or CD45RA- FOXP3high effector Treg (red) is shown.

Firstly identified in 1995 in mice as a suppressive CD4 T-cell subset constitutively expressing the IL-2 receptor alpha-chain (CD25) molecule (5), Treg have been subsequently identified in humans as a CD4 T-cell subset exhibiting *in vitro* suppressive properties and expressing high levels of CD25 (6–10). Unfortunately, the inducible nature of CD25 expression during T-cell activation on conventional T cells renders this molecule unsuited for Treg identification during immune-activation. Shortly thereafter, the forkhead box P3 (FOXP3) transcription factor was identified as an essential and specific factor for Treg development and function (11–13). While FOXP3 is to date the most specific marker for Treg identification in mice, in humans the situation is more complex, as the expression of FoxP3 is also observed in some conventional CD4+ CD25− T cells upon activation (14). Finally, it has been shown that human CD4+FOXP3+CD25high cells express lower levels of CD127, the alpha-chain of the IL-7 receptor, when compared with their FOXP3-counterpart (15–17). The combination of the CD25 and CD127 surface markers with or without intra-nuclear staining for FOXP3 expression has thereafter been widely employed to identify CD4+ Treg cells (Figures 1A–C). Sorting of Treg cells has greatly benefited from the combination of high CD25 and low CD127 expression. However such an approach also presents drawbacks: conventional non-Treg CD4 T cells down-regulate CD127 expression during activation while they up-regulate CD25. It is therefore likely that CD127 and CD25 expression cannot accurately discriminate *ex vivo* Treg cells from activated T cells in situations of immune-activation such as HIV infection (18). In conclusion, Treg identification in context of chronic activation such as HIV infection, still suffers from the lack of indisputable markers that can unequivocally distinguish Treg from effector cells.

### Treg susceptibility to HIV infection and suppressive properties

Several studies have shown *in vitro* that Treg cells are highly susceptible to HIV infection (19–22). Moreover, Treg susceptibility seems to differ depending on the HIV type 1 strain, Treg being less susceptible to R5 viruses compared with effector T cells (22). Interestingly, Tran et al. suggested that Treg could represent a preferential cellular reservoir of viral infection (21). Treg suppressive capacity does not seem to be affected by HIV infection as Treg isolated from acutely (23), chronically viremic (24, 49) infected patients, or HIV controllers (24, 49) suppress effector T cells proliferation as efficiently as Treg isolated from healthy donors.

### Treg quantification in HIV infection

Regulatory T cells quantification in HIV infection remains controversial in part because of the aforementioned lack of homogeneous and reliable specific markers to identify human Tregs. A second hurdle arises from the strategy used to quantify Tregs. Because CD4 depletion is the pathogenic hallmark of HIV infection and CD4 counts decline during disease progression, determining Treg percentages among CD4 T cells or Treg counts does not provide similar information and thus participates to the uncertainties concerning Treg quantification. Both quantifications have their advantages and drawbacks. Percentages allow analyzing relative proportion of Tregs among CD4 T cells regardless the intensity of CD4 T-cell depletion associated to HIV infection. Conversely, Treg numbers allow evaluating potential bias in effector non-CD4 T-cell/Treg ratio since Treg suppression not only affects CD4 T cells but also effector CD8 T cells and innate cells. Interestingly, these two analyses led to different observations: Treg numbers are essentially reduced during HIV infection, but Treg are preferentially preserved compared to conventional CD4 T cells thus diversely impacting Treg percentages among CD4 T cells. Extreme care should be taken when interpreting these opposing data. Increasing Treg percentages among CD4 T cells suggest increasing Treg mediated suppression capacities against CD4 T cells, but reduced Treg numbers suggest reduced suppression capacity on other cellular targets (CD8 T cells, NK cells, dendritic cells). It thus seems relevant to provide both sets of information to finely dissect the global impact of Treg during HIV infection. Such particular feature of Treg alteration during HIV infection may provide an important rationale to understand diversity of Treg mediated effects. Finally, a third major aspect participating to the discrepancies obtained is represented by the diversity of HIV clinical stages rendering universal conclusion difficult to be drawn. Exhaustive analyses addressing the different immunological and virological status of HIV patients finally allowed some consensual conclusions to be formulated. During primary HIV infection (PHI), decreased Treg percentages (23, 25, 26) and absolute numbers (26) have been described, although results differ depending on the staining strategy (25). In chronically infected viremic patients, Treg percentages are shown to be consistently increased while Treg absolute numbers consistently decreased (24, 26–35). During efficacious ART Treg percentages have been shown to progressively decrease to normal levels (33, 36–40) while Treg counts increase progressively in parallel with total CD4 counts (24, 33, 37–39, 41). Interestingly this effect is reversed upon ART interruption (42). Studies of Treg levels in HIV controllers, a subpopulation of patients who spontaneously control viral loads (43), revealed alternately unchanged (20, 24, 39, 44), increased (45), or reduced (26, 46) percentages compared to healthy donors. Treg absolute numbers have been reported to be either unchanged (39) or decreased (26) in HIV controllers compared to healthy individuals. Importantly, analyses of Treg homeostasis were also performed apart from peripheral blood and notably in gastrointestinal mucosa which represents a major site of viral replication and CD4 T-cell depletion, and thus represents a central site involved in HIV infection pathogenesis. Few studies investigating Treg levels in the intestinal tract demonstrated that during viremic HIV infection a global CD4 T-cell loss takes place in gastrointestinal mucosa with preferential preservation of Treg, leading to a relative increase in Treg proportion (24, 47–49). Such a process is reversed by efficient ART during which restoration of normal Treg levels in gastrointestinal tract is observed (24, 47–49). Collectively, data obtained from intestinal tract thus corroborated data obtained from peripheral blood showing a reduction of Treg numbers in peripheral blood and gut mucosa during viremic stages, although Tregs appeared preferentially preserved among CD4 T cells at both sites.

### Impact on HIV progression

Several studies tried to evaluate *in vivo* Treg role on HIV infection by correlating Treg percentages or numbers to different canonical parameters of HIV disease, i.e., CD4 count, viral load, and activation profile. Most of published studies agree in reporting a positive correlation between absolute numbers of Treg and CD4 counts (26, 28, 31, 37, 39, 50–54) and a negative correlation between Treg percentages and CD4 counts (27, 29, 31, 34, 37, 39, 55, 56). One may discuss the relevance of correlation between CD4 counts and Treg counts because Treg are a CD4 T-cell subset. Interpretations were thus mostly drawn from correlation with viral load and/or activation profile. Viral load has been reported mostly to be positively correlated to Treg percentages (29, 39, 40, 55, 57) and negatively correlated with Treg numbers (31, 39, 51, 58). These observations led to investigate a direct impact of Treg on HIV-specific CD4 and CD8 T-cell responses. It has been shown that presence of CD4+ CD25+ cells during *in vitro* HIV-specific stimulation led to decreased HIV-specific CD4 and CD8 responses (20, 21, 23, 50, 51, 59–66). Importantly, Treg have been reported to suppress both cell proliferation and effector molecules production in response to HIV. Recently, Elahi and coworkers reported the interesting finding that Treg cells differentially suppress HIV epitope proliferation of CD8+ CTLs depending on HLA alleles restriction, epitope-specific CD8+ CTLs restricted to the protective HLA allele groups HLA-B∗27 and HLA-B∗57 being not susceptible to Treg mediated suppression (63). Collectively, published results suggest that a dominant mechanism of suppression by Treg could reduce *in vivo* antiviral responses participating to the incapacity to eradicate HIV infection. However, *ex vivo* correlation studies failed to detect any association between global Treg levels and HIV-specific T-cell responses in terms of IFN gamma (IFN-γ) production in response to HIV peptides stimulation (24, 40, 44, 51) or HIV-specific CD8 T-cell activation (26).

Immune hyper-activation as revealed by expression of CD38 and HLA-DR activation markers at CD4 and CD8 cell surface is a negative prognostic factor associated with disease progression in HIV infection (67, 68). In order to investigate if Treg alteration played a role in this phenomenon, several studies have tried to identify a correlation between Treg level and lymphocytes activation. Unfortunately no clear conclusion can be drawn using expression of CD38 and HLA-DR expression analyses to evaluate CD4 and CD8 activation. Conflicting results have been reported concerning relationship of Treg with immune-activation in PHI depending on Treg identification strategy employed (23, 69). Similarly, no consensus has been reached in studies including patients during chronic viremic infection when either positive (29, 53), negative (25, 51, 70), or no correlations (32, 55) between Treg levels and immune-activation have been reported. The only stage of infection in which some conclusion can eventually be reached is represented by aviremic patients undergoing ART in which several studies concluded for an inverse correlation between Treg proportions and T-cell activation (36, 42). Based on these observations some authors speculated that Treg could be sufficient to control low residual T-cell activation in ART-treated patients, but insufficient to affect generalized immune-activation observed during primary or chronic viremic HIV infection (4). In this hypothesis, restoring Treg pool in ART-treated patients may thus constitute an interesting strategy ensuring Treg suppression without affecting viral load control. IL-2 recombinant injection induces significant increase in Treg numbers (111) although no clinical benefits were observed from restoration of Treg pools, casting doubts on the relevance of Treg compartment on the control of immune-activation (71). However, the Treg subsets preferentially increased upon IL-2 treatment are not fully characterized. It could be useful to consider manipulation of specific Treg subsets to provide beneficial impact. Collectively, these contrasting results reinforce the notion that Treg could be a double edged sword during HIV infection. On one side, they are detrimental as they inhibit HIV-specific immune response. On the other side, they could participate to the maintenance of immune-homeostasis by reducing non-specific chronic immune hyper-activation. Taking into account Treg heterogeneity may prove crucial to dissect these opposite effects of Treg mediated suppression during HIV infection.

## HIV Infection and Tregs Subsets

Similarly to other T-cells compartments, Treg population presents a high degree of heterogeneity both in humans and mice. Several markers allow the identification of phenotypically distinct Treg subsets, and controversies exist about which Treg subpopulations present peculiar functional characteristics. As described for conventional T cells, distinction between naïve and effector cells have been considered for Treg subsets. CD45RA or CD45RO expression has been notably considered to identify naïve and effector Tregs. Such strategy offers interesting insight on the biology of Tregs in HIV infection as discussed below, being an initial step that partially reflects the heterogeneity among Treg subsets. Other markers, such as HLA-DR or Inducible costimulatory molecule (ICOS), have been reported to identify cells at other stages of activation and, more importantly, seem to identify peculiar Treg cell subsets provided with specific mechanisms and effects of suppression. Finally, some molecules implicated in Treg suppressive function, such as CD39 and Glycoprotein A repetitions predominant (GARP), are employed to identify peculiar Treg subsets. Defining additional markers and/or combination will undoubtedly allow further refining of Treg subsets preservation during the course of HIV infection. Identification of Treg subsets using activation markers and their respective impact on HIV infection will be briefly presented when information is available.

### Subdivision in naïve and effector Treg subsets

Several studies have shown that CD45RA is an extremely useful marker for Treg identification when combined to CD25 (72, 73, 110) or FOXP3 (74). CD45RA expression allows the repartition of the FOXP3+ CD4 T-cell population in three subsets: (i) FOXP3low CD25low CD45RA+ cells, (ii) FOXP3high CD25high CD45RA− cells, and (iii) a FOXP3low CD25low CD45RA− population (74) (Figures 1B,C). FOXP3low CD25low CD45RA+ cells represents in human peripheral blood approximately 2–4% of CD4 T cells and 20–30% of “global” CD4+ FOXP3+ CD25high CD127low T cells. Accordingly to their “naïve” phenotype, CD45RA+ FOXP3low CD25low Tregs constitute the great majority of CD25 or FOXP3 expressing CD4 T cells in cord blood (73, 74). In classical *in vitro* suppressive assays CD45RA+ FOXP3low naïve Treg cells efficiently suppress effector T-cell proliferation (74). Interestingly during activation these cells actively proliferate, are highly resistant to apoptosis and convert to a CD45RA-CD45RO+ phenotype (74). It is important to note that early studies of human Treg based on the CD25high gating strategy initially proposed by Bacher-Allen (6, 75) inadvertently excluded the CD25low naïve Treg population from the analysis.

CD45RA− FOXP3+ CD4+ T cells include as mentioned two phenotypically and functionally distinct cellular subpopulations: (i) a FOXP3low CD25low CD45RA− cytokine-secreting cell population which lacks suppressor activity (ii) a suppressive effector FOXP3high CD25high CD45RA− Treg population (74). Such an analysis presents the major advantage of allowing the exclusion of the FOXP3+ non-Treg contaminating cells, which are included when the classical FOXP3+ CD25hi CD127low gating strategy is employed. FOXP3high CD25high CD45RA− effector Treg efficiently suppress conventional effector T-cell responses *in vitro* but, in contrast to CD45RA+ naïve Treg, are highly susceptible to apoptosis and mostly die while exerting their suppressive function (74). CD45RA+ naïve Treg are able to differentiate into FOXP3high CD25high CD45RA− effector Treg upon *in vitro* and *in vivo* activation (74). However, whether the FOXP3high CD25high CD45RA− effector Treg pool is entirely represented by activated thymic derived Treg or can be composed by peripherally differentiated induced Treg (iTreg) still remains unknown.

#### CD45RA+FOXP3low naïve Treg cells

CD45RA+ FOXP3low CD25low Treg counts have been reported to be significantly reduced in HIV-infected patients when compared with healthy donors (26, 76). When different HIV diseases stages are taken into account naïve Treg counts reduction seems to be exclusively restricted to the PHI phase, while no difference is observed when viremic and aviremic chronically infected patients are considered (26). The majority of naïve CD45RA+ FOXP3low CD25low Treg also express CD31 (platelet endothelial cell adhesion molecule-1, PECAM-1), a cell surface marker identifying recent thymic emigrants. Proportions of CD31+ among naïve Treg are not affected during acute or chronic HIV infection indicating preserved Treg thymic differentiation (77). Naïve Treg express high levels of the HIV co-receptor CXCR4 while CCR5 is barely detectable at their surface (76–78). *In vitro* experiments have demonstrated that CD45RA+ naïve Treg are more susceptible to HIV infection when compared to conventional CD45RA+ naïve CD4 T cells (76, 78). Accordingly to their phenotype naïve Treg were more susceptible to *in vitro* infection when CXCR4-tropic strain (HIV-1 IIIB) was used rather than CCR5-tropic strain using HIV-1 BaL (76).

Regarding the association of naïve Treg cell numbers and parameters of disease in HIV infection, naïve Treg cell numbers positively correlate with CD4 count in both healthy donors and HIV-infected patients (26, 31, 76) independently from the stage of the disease. No association (76) or only weak inverse correlation (26, 31) between levels of HIV RNA levels and number of naïve Tregs have been reported. Finally, naïve Treg cell numbers correlate neither with global CD8 T-cell activation nor with HIV-specific CD8 T-cell responses (26).

Globally, current evidence indicates that naïve Treg subset is minimally affected during HIV infection being altered exclusively during early phases of infection (primary infected patients). *Ex vivo* correlation analyses indicate only marginal role of naïve Treg on HIV infection.

#### CD45RO+ FOXP3high CD25high effector Treg cells

No differences (26, 76) or increase (77) were reported in proportions of effector Treg among CD4 T cells during viremic chronic infection. In contrast, viremic chronically HIV-infected patients present a significant reduction in effector Treg cell counts when compared with healthy donors (26, 76), and this phenomenon was observed in other settings of disease including patients during PHI, aviremic patients under antiretroviral treatment, and HIV controllers (26). Therefore, effector Treg depletion appears to take place early during HIV infection and to persist during chronic phases of infection, while ART seems not to be able to restore the effector Treg pool. However, further studies analyzing cohorts followed up longitudinally may be needed to determine the effects of ART on reconstitution of the effector Treg subset. Accordingly to its expression on recent thymic emigrants, CD31 is barely detectable at effector Treg surface (74). Interestingly, Zhou and coworkers showed increased proportions of CD31 expressing effector Treg in both acutely and chronically HIV-infected patients suggesting higher conversion from a naïve to an effector Treg phenotype during HIV infection (77).

Phenotypic analysis of effector Treg revealed high levels of expression of the HIV co-receptor CCR5 while CXCR4 is expressed at lower levels at effector Treg surface when compared to naïve Treg cells (76, 77). Such a differential pattern of HIV co-receptor expression between naïve and effector Treg cells suggests potential differences in viral strains infection susceptibility. Indeed, effector Treg were more susceptible than naïve Treg to *in vitro* HIV infection by CCR5-tropic HIV-1 BaL while naïve and effector Treg were similarly susceptible to CXCR4-tropic HIV-1 IIIB *in vitro* infection (76).

Some conclusions can be drawn from correlation analyses regarding the role played by effector Treg in HIV infection. Effector Treg numbers positively correlate with CD4 counts in healthy donors and such a correlation is lost in chronically HIV-infected individuals (26, 76) presumably reflecting a preferential loss of the effector Treg subset. Lack of correlation between effector Treg numbers and CD4 counts was similarly found in patients during PHI and in individuals under efficacious ART (26), suggesting an early impairment in effector Treg homeostasis during HIV infection which is not restored by ART. Interestingly, the correlation was observed in HIV controllers, indicating a preserved effector Treg pool in this peculiar patient population. No correlation between effector Treg counts and HIV viral load (26, 76) or global CD8 T-cell activation (26) has been reported. Aiming to determine the role of effector Treg in modulation of HIV-specific immune-responses, we found an inverse correlation between effector Treg counts and both HIV-specific CD8 activation and interferon gamma production by CD8 upon stimulation by HIV peptides (26). These *ex vivo* results suggest a dominant suppression exerted by Treg on HIV-specific CD8 T-cell responses potentially participating to the incapacity to control the virus. Globally, available data indicate a preferential, precocious, and long-lasting effect of HIV infection on the effector Treg compartment. Analyses of association between Treg subsets and HIV disease parameters, while failing to detect any potential link with the naïve Treg subset, point to a deleterious effect of effector Treg in HIV infection pathogenesis. Further studies will eventually confirm the dominant suppressive role exerted by effector Treg on HIV-specific immune-responses currently suggested by observational data. Surprisingly, no or low correlation was detected between Treg and immune-activation so far. One may discuss the accuracy of the immune markers selected to determine such association. Secondly, specificity of effector Treg cells recovered during HIV infection also introduces heterogeneity. Demonstrating whether residual effector Treg are specifically targeting HIV related epitopes may further ascertain or infirm the specific role of Treg on HIV-specific responses.

### Additional subdivision among effector Tregs

Although highly altered in numbers by HIV infection, effector Tregs recovered from HIV-infected patients exhibited various phenotypic profiles. The relative susceptibility to HIV infection of each effector Treg subsets and their respective suppressive capacity remains to be further elucidated.

#### HLA-DR

MHC-II expression identifies a population that represents about 20–30% of human circulating Treg cells (6, 79). *Ex vivo* isolated HLA-DR+ Treg cells suppress responder T-cell proliferation and cytokine secretion more efficiently and more rapidly than HLA-DR-Treg cells (79). Importantly, all HLA-DR+ Treg cells are part of the effector FOXP3highCD45RA− compartment (74) (Figure 1D) of which they seem to constitute a terminally differentiated subset [reviewed in (80)].

Little is known about the effects of HIV infection on HLA-DR+ terminally effector Tregs or about the role exerted by this subset in the pathophysiology of the disease. Higher proportions of HLA-DR expressing Treg are present in chronically viremic HIV-infected patients when compared to healthy donors (81, 82) and Treg from patients presenting higher viral loads express higher levels of HLA-DR (81). ART fails to normalize HLA-DR+ Treg proportions (82). Unfortunately, no information about HLA-DR+ terminally effector Treg counts is available and further studies may be needed to determine whether the reported alterations merely reflect a phenotypic modification linked to the activation status or whether HIV infection directly alters HLA-DR+ terminally effector Treg homeostasis. Correlation analysis demonstrated an inverse correlation between proportions of HLA-DR+ Tregs and CD4 counts (81, 83), while a positive correlation was reported between percentages of HLA-DR positive cells among Treg and viral load (83) or CD4 and CD8 T-cell activation as revealed by HLA-DR or CD38 expression (81). Once more it is currently impossible to ascertain whether these relationships simply reflect HLA-DR up-regulation at Treg surface as a result of the global immune-activation observed during HIV or identify terminal effectors HLA-DR+ Treg as players in HIV physiopathology.

#### Ki67

Intracellular Ki67 staining identifies an actively proliferating fraction of Treg cells. In both mice and humans the percentage of Ki67 cells among FOXP3+ cells is higher than the percentage among conventional FOXP3− CD4 T cells (84). This is in accordance with their more activated profile. Notably, all cycling Ki67+ Treg cells are part of the effector FOXP3highCD45RA− compartment (74) (Figure 1D). During HIV infection, higher proportions of Ki67+ Treg are present during acute (77) and chronic viremic (33, 77, 83, 85) phases of infection. Longitudinal studies indicate that ART leads to normalization of Ki67+ Treg percentages (77, 83). Long term non-progressors present similar proportions of Ki67+ Treg as healthy donors (83) while no data are available in HIV controllers. Higher proportions of Ki67+ Treg seem to be associated with disease progression during chronic HIV infection as percentages of Ki67+ Treg correlate negatively with CD4 counts (33, 83) and positively with viral loads (33, 83). Collectively, higher levels of Ki67+ Treg have been associated with more advanced disease although studies addressing Ki67+ Treg counts and eventually analyzing HIV controllers could provide further insights into the role of this Treg subset in HIV physiopathology.

### Other subsets

#### CD39

CD39, also referred to as ENTPD-1, is a member of the ectonucleotidase triphosphate diphosphohydrolase family which hydrolyzes extracellular ATP and adenosine diphosphate (ADP) into adenosine monophosphate (AMP). Through CD39, Tregs can generate the inhibitory molecule adenosine which suppresses effector T cells by binding to the adenosine receptor 2A at their surface (86). While murine Treg mice globally express CD39 at their surface (86, 87), CD39 expression in human Treg is restricted to a subset of CD45RO expressing cells mostly co-expressing HLA-DR (87). Proportions of CD39+ Treg are significantly increased in HIV-infected patients, included chronic viremic patients, antiretroviral treated individuals, and long term non-progressors (39, 65, 82). Interestingly, HIV controllers present proportions of CD39+ Treg similar to healthy donors (39). Longitudinal analysis confirmed that antiretroviral treatment fails to normalize proportions of CD39+ Treg (39). *In vitro* suppression assays revealed that the suppressive effect of Treg on cytokines production of Gag-stimulated CD8+ T cells is partially reversed by the addition of CD39 blocking mAb (65), pointing to a role for CD39 in Treg suppression of HIV-specific responses. Interestingly, CD39 is also involved in Treg control of HIV viral replication (64). CD39 expression on Treg correlates negatively with CD4+ T-cell count and positively with viral load and T-cell activation in HIV-1 positive subjects (39, 65). Globally, current evidence points to a major role for CD39 expression on Treg, participating to Treg mediated suppression of HIV-specific responses and disease progression.

#### Inducible costimulatory molecule

Inducible costimulatory molecule is a costimulatory molecule involved in cell activation that is expressed on effector/memory T-cell subsets. In mice, ICOS represents an activation marker at the Treg surface. *Ex vivo*, its expression identifies a sizable population which represent about 10–20% of CD4+FOXP3+ T cells isolated from secondary lymphoid organs. Whether ICOS expression exerts any function in Treg activity is still unknown. In humans differential expression of ICOS has been shown to delineate two different subsets of Treg cells in the peripheral blood (88). Interestingly, these two phenotypically distinct subsets present differences in their suppressive capacity and in the mechanisms of action employed: ICOS− Treg suppression is mainly mediated by TGF-β while ICOS+ Treg suppression relies predominantly on IL-10 (88). Higher proportions of ICOS expressing Treg have been reported in several populations HIV-infected patients, including viremic chronically infected patients, antiretroviral treated individuals, and HIV controllers (39). Longitudinal analysis revealed significant decrease of ICOS expressing Treg following ART treatment (39).

#### Glycoprotein A repetitions predominant

Glycoprotein A repetitions predominant (or LRRC32) is a transmembrane protein selectively expressed by activated Treg but not conventional CD4 T cells (89–91). *Ex vivo*, GARP identifies a subset of activated FOXP3+ T cells with high suppressive capacity. While barely expressed on CD45RA+ naïve Treg, GARP is promptly up-regulated upon *in vitro* TCR-stimulation on both naïve and total Treg cells, while no expression is detected on activated conventional T cells (90). Moreover, human CD4 T cells in which FOXP3 expression was induced by activation in the presence of TGF-β failed to express GARP, leading to the hypothesis that GARP could be employed to identify *bona fide* Tregs (90). Two studies comparing GARP+ Treg proportions in healthy donors and HIV-infected individuals, failed to detect any significant difference (82, 90). This result was in discordance with the increase in proportions of FOXP3+ CD4 T cells reported in the same studies, leading to the hypothesis that a portion of FOXP3+T cells detected during HIV infection are possibly recently activated cells and/or iTreg. Further studies combining GARP expression with effector Treg and iTreg identification strategies will eventually clarify this issue.

### Subdivision in extrathymically induced or adaptive Tregs

In addition to their activation profile, Treg has also been dissected in two subsets: natural Tregs originating from the thymus and extrathymically iTregs or adaptive Tregs generated in the periphery under a variety of conditions through conversion from naïve conventional CD4+ cells. Several mechanisms have been implicated in induced Foxp3+ Treg (iTreg) peripheral generation, including cytokines (TGF-β, IL-2) and metabolic pathways (tryptophan metabolism, retinoic acid).

### *Ex vivo* iTreg identification

*Ex vivo* quantification of iTreg during HIV is limited by the unavailability of a specific marker for identification of *bona fide* iTreg. Several markers have been proposed for iTreg identification but their specificity in distinguishing natural thymic derived Treg from peripherally differentiated iTreg is still a matter of debate.

#### Helios

The transcription factor Helios, a member of the Ikaros transcription factor family, has been reported to be expressed by 100% of CD4+CD8−Foxp3+ thymocytes and about 70% peripheral Foxp3+ T cells in mice and humans (92). Interestingly, murine or human naïve T cells acquiring Foxp3 expression upon *in vitro* TCR-stimulation in the presence of TGF-β failed to express Helios, suggesting that absence of Helios expression could be employed to identify peripherally differentiated iTreg (92). However, subsequent reports showed that depending on the stimulation conditions, Helios could be induced in parallel with Foxp3 in iTreg (93–95). Moreover, Helios was reported to represent a T cell activation and proliferation marker thus being independent from Treg lineage commitment (96). Finally, natural Treg recent thymic emigrants have been shown to contain a fraction of Helios negative cells (97). Regardless its limited reliability as a maker differentiating nTreg from iTreg, Helios expression allows the identification of a subset of Treg cells presenting some peculiar characteristics. Murine Helios+ Treg express higher levels of CD103 and GITR at their surface and produce higher levels of TGF-β (94). Interestingly, murine and human data indicate that Helios+ Treg are relatively over-represented in tumors, pointing to this cell subset as a potential target for immune-modulating strategies (94, 98, 99). Currently, no information is available concerning Helios expression on Treg cells during HIV infection. Further studies will eventually define whether HIV infection differentially affects Helios+ and Helios− Treg subsets homeostasis and whether these subsets play a distinct role on HIV pathophysiology.

#### Neuropilin-1

Neuropilin-1 (NRP1) is a semaphorin III receptor participating in axon guidance, angiogenesis, and involved in the immunological synapse, which has been recently reported in mice to be expressed at high levels on thymic derived nTreg cells but not on peripherally generated iTreg (100, 101). Importantly, NRP1 expression remains stable on NRP1 positive or negative Treg subsets upon TCR mediated or lymphopenia induced cell activation and proliferation while environmental inflammatory stimuli have been shown to modulate NRP1 expression (101). Therefore, NRP1 expression has been suggested as a potential marker distinguishing nTreg from iTreg, at least in steady state conditions. Whether these results obtained in the murine system can be translated to human cells still remains to be addressed. In humans, NRP1 seems to be expressed exclusively by a subsets of lymph-node resident Treg subset (102) while Treg isolated from human peripheral blood fail to express significant levels of NRP1 (102, 103). One study performed in HIV infection assessed proportions of Neuropilin-1 expressing CD4 T cells and correlation with other Treg markers and failed to detect any significant difference between healthy donors and viremic or aviremic antiretroviral treated HIV+ patients (104).

### HIV infection favors peripheral Treg induction

Numerous studies suggest an effect of HIV on iTreg cells generation mainly through modulation of antigen presenting cells (APC) tolerogenicity. Plasmacytoid dendritic cells (pDCs) represent a crucial subset of APC involved in antiviral immunity and a major target of HIV infection. Through Toll-like receptor 7 stimulation, HIV modulates pDC activation by simultaneously inducing type I IFN production and up-regulation of indoleamine 2,3 dioxygenase (IDO) expression (105, 106). IDO is an enzyme involved in tryptophan catabolisms which exerts immuno-modulatory functions by inhibiting T-cell proliferation and inducing iTreg peripheral generation. HIV-activated human pDC induce the peripheral generation of iTreg (106) through IDO up-regulation. Consequently, iTreg induced by HIV-activated pDC modulate myeloid dendritic cells (mDC) maturation and function partially through cytotoxic T-lymphocyte antigen (CTLA)-4 engagement, inhibiting their maturation and inducing IDO expression (106, 107). CTLA-4-conditioned mDC can in turn induce Treg differentiation in an IDO-dependent manner (107). Whether HIV could directly modulate mDC capacity to generate iTreg still remains unclear. Lymph-node resident mDC from viremic but not ART-treated HIV-infected subjects induce iTreg differentiation phenotype of normal allogeneic T cells (108). Accordingly, preclinical data in the SIV infection model indicate that mature splenic or mesenteric mDCs from SIV-infected animals are significantly more efficient at inducing Treg than mDCs from uninfected animals (82). However, experimental evidence indicates that *in vitro* infection of mDC with CCR5-utilizing virus or even simple exposure of mDC to inactivated HIV significantly impairs their ability to induce iTreg differentiation (109).

## Concluding Remarks

**Figure 2:**
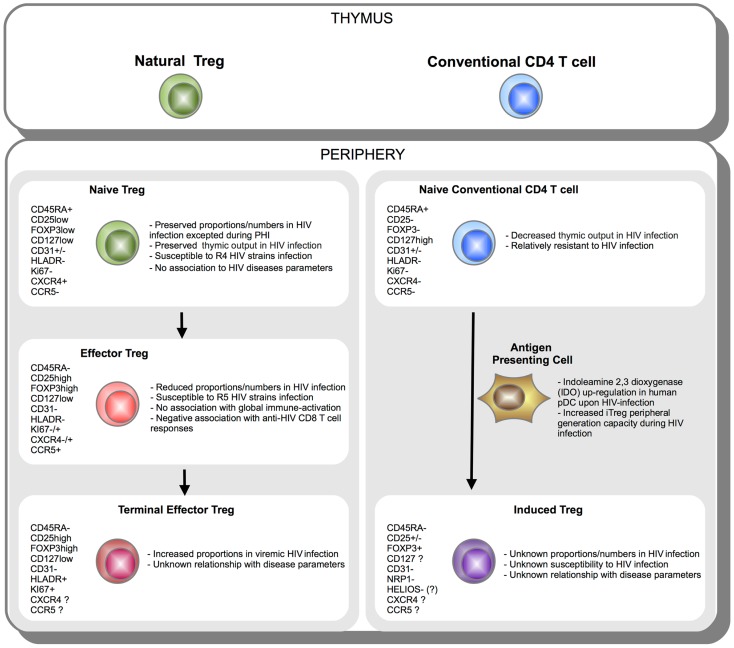
**Schematic representation of Treg subsets during HIV infection**. CD4 T cells originate in the thymus as Natural FOXP3+ Treg or conventional FOXP3− T cell. Once in the periphery, natural CD45RA+ FOXP3low naïve Treg cells further differentiate into effector CD45RA-FOXP3hi and terminal effector CD45RA-FOXP3hiHLADR+ Tregs (*left panel*). On the other side, upon activation under specific tolerogenic conditions such as tolerogenic antigen presenting cells (APC) expressing indoleamine 2,3 dioxygenase (IDO), conventional naïve FOXP3− CD4 T cells can convert extrathymically into induced FOXP3+ Treg (iTreg) (*right panel*). Phenotypic markers expressed during Treg subsets differentiation or peripheral iTreg conversion are indicated. Essential aspects of Treg subsets relationship with HIV infection are summarized.

Human Treg quantification, especially in contexts of chronic immune-activation such as HIV infection, still remains uncertain essentially because of limitations in identification strategies. We discussed how dissecting Treg heterogeneity provided additional insights on the biology of Treg during HIV infection. A schematic representation of Treg subsets during HIV infection is provided in Figure 2. Currently two main strategies are used to classify and characterize Treg subsets. In accordance with classification established for conventional T cell, analyses of “naïve” and effector Tregs have been considered. Importantly, naïve and effector Treg discrimination led to better identification of Treg by limiting contamination by Foxp3low non-Treg cells. Use of classical markers of T-cell activation (CD45RA/RO, HLA-DR, Ki67, ICOS) or use of markers more specific to Treg subsets have been considered. Such distinction among naïve and effector Treg subsets allowed unveiling differences in HIV infection susceptibility and homeostatic behavior during HIV infection. We and others reported a preferential role for effector Treg compartment in immune-regulation during HIV infection. This two-step discrimination allows approaching Treg heterogeneity, but still remains incomplete. High heterogeneity presumably stands among effector cells and remains to be further investigated. A second classification is based on the identification of natural versus peripherally iTregs. Whereas understanding immune-regulation developing during HIV infection may be greatly improved from the precocious analysis of iTregs, the current lack of a reliable marker to identify these cells currently precludes consensual conclusions to emerge. Despite major advances in recent years, this is the early stage of Treg heterogeneity analysis in the context of HIV infection. Further studies will hopefully identify deleterious and beneficial Treg subsets and allow designing accurate restoration strategies to reduce chronic immune inflammation during HIV infection.

## Conflict of Interest Statement

The authors declare that the research was conducted in the absence of any commercial or financial relationships that could be construed as a potential conflict of interest.
